# Influence of Weather Conditions on the Onset of Spontaneous Pneumothorax in the Region of Sousse (Tunisia): Analysis of Time Series

**DOI:** 10.1155/2019/1793973

**Published:** 2019-05-07

**Authors:** Sana Aissa, Maher Maoua, Salsabil Selmi, Wafa Benzarti, Imen Gargouri, Ahmed Abdelghani, Abdelhamid Garrouche, Abdelaziz Hayouni, Mohamed Kahloul, Walid Naija, Nejib Mrizek, Mohamed Benzarti

**Affiliations:** ^1^Pneumology Department, University Farhat Hached Hospital, Sousse, Tunisia; ^2^University of Sousse, Faculty of Medicine Ibn Jazzar, Sousse, Tunisia; ^3^Occupational Medicine Department, University Farhat Hached Hospital, Sousse, Tunisia; ^4^Anesthesia Intensive Care Unit, University Sahloul Hospital, Sousse, Tunisia

## Abstract

**Introduction:**

Weather conditions were implicated in the onset of spontaneous pneumothorax (SP).

**Aim:**

Investigate the influence of weather conditions on the onset of SP.

**Methods:**

A total of 200 patients with SP in Sousse (Tunisia) were enrolled in the study between January 2010 and December 2014. An analysis of two time series (meteorological data and pneumothorax cases) was performed. Data on weather conditions were collected daily throughout the 5-year period.

**Results:**

A comparison of the mean temperature between days with and without SP showed significantly higher temperatures during the days with SP. A decrease of 1% in the relative humidity one day lag (D-1) was associated with an increase in the risk of SP by 1.6% (p=0,02). The occurrence of clusters was associated significantly with higher temperature averages on the same days. This same observation was made regarding the mean duration of sunshine two days before the cluster onset (p = 0.05). The occurrence of storms two days before clusters was also significantly associated with a risk multiplied by 1.96.

**Conclusion:**

There was a correlation between clusters of spontaneous pneumothorax and weather conditions in the region of Sousse-Tunisia.

## 1. Introduction

### 1.1. Background and Rationale

Spontaneous pneumothorax (SP) is frequent and its incidence is increasing. It is a respiratory disease that may be severe [1, 2]. Pneumothorax is defined as the entry of air into the pleural space. Spontaneous pneumothorax may be primary, occurring in patients without clinically apparent lung disease, or secondary, arising as a complication of antecedent lung disease [3–6]. Exact pathogenesis is partially known. Most authors state that a spontaneous pneumothorax results from the rupture of a bubble of blebs or an emphysema bubble that is localized in the visceral pleura [7]. The rupture of this bubble occurs when a transpulmonary pressure gradient is present [8, 9]. However, little is known about the factors that trigger the rupture of the blebs and bullae that occur in SP. Previous studies have suggested that seasonal variations and meteorological factors may possibly influence the incidence of SP. While some studies have linked atmospheric pressure and decreasing humidity with an increased incidence of SP, others have reported no association between the occurrence of SP and climatic conditions, such as changes in atmospheric pressure, temperature, and humidity.

Some authors have shown that the occurrence of a spontaneous pneumothorax can be influenced by certain climatic variations [9]. Other studies have highlighted the impact of other factors, such as scuba diving, exposition to loud music, or a roller coaster ride [10].

Climatic changes in atmospheric pressure, temperature, or humidity may influence the incidence of pneumothorax [11].

### 1.2. Objective

The aim of the present study is to explore relationships between the occurrence of spontaneous pneumothorax and the variation of meteorological parameters (mean temperature, atmospheric pressure, relative humidity, duration of sunshine, rainfall, and storms) among adults in Sousse (Tunisia) during a 5-year period. This study was designed to investigate the relationship between the occurrence of clusters and the variability of meteorological parameters.

## 2. Methods

### 2.1. Study Design

We conducted a study of 2 time series (meteorological parameters and cases of pneumothorax) over a period from January 1^st^, 2010 to December 31, 2014. There was no need for an ethics committee approval, since the lack of patients' contact or intervention.

### 2.2. Setting

Patients hospitalized with a diagnosis of spontaneous pneumothorax between 1 January 2010 and 31 December 2014 were enrolled this study. Patients were treated for spontaneous pneumothorax in the pneumology department, in Farhat Hached Hospital in Sousse, and Anesthesia Intensive Care Unit in Sahloul Hospital in the region of Sousse during the five years of the study representing the two public structures supporting patients for this pathology.

### 2.3. Participants

Only patients with spontaneous pneumothorax, whether idiopathic, secondary, or relapsing, residing in the governorate of Sousse during the week preceding pneumothorax were included.

Patients with induced pneumothorax (posttraumatic or iatrogenic) and those transferred from another geographic area not under the same meteorological conditions were excluded.

### 2.4. Variables

For the purposes of the study, two separate synoptic sheets were used: (1) a first sheet relating to the daily meteorological data which included the following variables: date, season, mean temperature, mean temperature one day lag (D-1), average temperature two days lag (D-2), average relative humidity, average relative humidity one D-1, average relative humidity at D-2, mean atmospheric pressure, mean atmospheric pressure at D-1, mean atmospheric pressure at D-2, average duration of sunshine, average duration of sunshine at D-1, average duration of sunshine at D-2, the occurrence of thunderstorms, occurrence of precipitation, occurrence of pneumothorax, and daily number of pneumothorax and (2) a second sheet relating to the medical data including identification of the patients, sex, age, smoking status, localization of the pneumothorax, medical history, date of occurrence of pneumothorax, evolution, and recurrence.

### 2.5. Data Sources and Measurements

Meteorological data were obtained from the National Institute of Meteorology (NIM) from the meteorological station Monastir-Airport for the agglomeration of Sousse located at 14 Km and this distance allows the extrapolation of measured data to the region of Sousse with good validity [12]. They concern, namely, the average daily temperature in degrees (°C), the average relative humidity (%), the average duration of sunshine (hours), the average atmospheric pressure expressed in hectopascal (hPa), the occurrence of thunderstorms, and the occurrence of precipitation (rainfall).

In addition to patients, a time series of all days of the study period was compiled with daily averages of all measurable meteorological variables of interest as well as daily weather events.

#### 2.5.1. Bias

A selection bias is noted. In fact, patients included have been identified in the university hospitals where the management of SP is carried out in the region of Sousse. Patients with SP in private clinics were not included.

#### 2.5.2. Study Size

During the study period, the study included convenience retrospectively consecutive patients treated for spontaneous pneumothorax.

### 2.6. Quantitative Variables

In our study, the dependent variable was the occurrence of pneumothorax, approached in two different ways: the occurrence of one or more episodes of pneumothorax, at a given date, which was considered as a positive response (presence of pneumothorax) and a count data, corresponding to the number of pneumothorax per day during the study period (0,1, 2…).

On the other hand, a “cluster” was defined by a period less than or equal to 3 days between two episodes of spontaneous pneumothorax. Every day between the first episode and the last episode of pneumothorax was enrolled in this cluster.

The independent variables were the meteorological data. These data were recorded for all days included in the study. Associations between pneumothorax events and meteorological data of the same day (D0), one day lag (D-1), and two days lag (D-2) were studied.

### 2.7. Statistical Methods

In the descriptive study, frequencies and percentages were calculated for the qualitative variables as well as means, standard deviation, medians, quartiles, and range of extreme values for quantitative variables.

For the comparison of means, Student's “t” test was used for paired or independent series averages. Anova's one-factor test was applied for the comparison of several averages.

The frequency comparison was performed by the Pearson's Chi-square test. The study of the relationship between two quantitative variables was performed by the Pearson's correlation coefficient.

For time series analysis, the Poisson generalized linear model (GLM) (Log-linear) was used with, as a dependent variable, a count data represented by the number of pneumothorax per day, after verification of the Poisson distribution. A multiple binary logistic regression was performed, with, as a dependent variable, the absence or presence of pneumothorax at a given date.

For all statistical tests, the significance level of p-value was set at 0.05. All statistical analyses were performed using SPSS 20.0 software (IBM Corp. Released 2011. IBM SPSS Statistics for Windows, Version 20.0. Armonk, NY: IBM Corp).

## 3. Results

### 3.1. Participants

Throughout the study period, 200 spontaneous pneumothoraxes were collected.

### 3.2. Descriptive Data

The study population was relatively young with a mean age of 28 ± 17.11 years and the most represented age group was between 21 and 40 years.

Of the 200 patients admitted for SP, 191 (95.5%) were male patients. Smoking was noticed among 162 (81%) patients. In regard to comorbidities, 1% of patients had a diagnosis of asthma, 6% had chronic obstructive pulmonary disease (COPD), and 0.5% had interstitial lung disease. SP was idiopathic among 151 (75.5%) patients and secondary among 49(24.5%). SP was total in 73.5% of cases and partial in 26.5% of cases.

Table 1 shows a summary profile of the study population.

Table 2 shows the means for five climatic variables during the five-year study period: temperature (20.6°C), relative humidity (65.55 %), atmospheric pressure (1015.43 hPa), rainfall (321mm), and duration of sunshine (11,97 hours).

### 3.3. Outcome Data

#### 3.3.1. Seasonality of Spontaneous Pneumothorax

**Figure 1:**
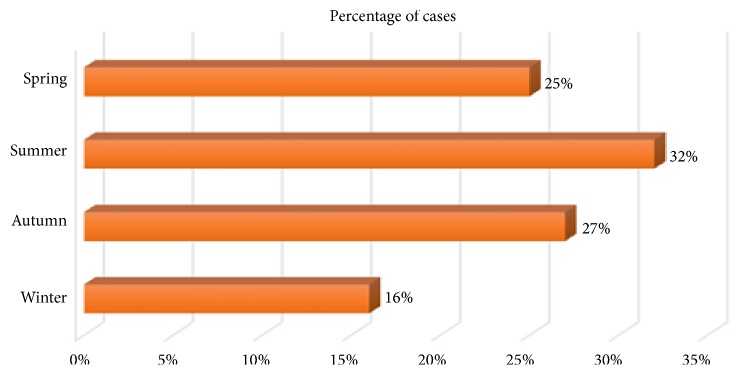
Distribution of cases according to seasons.

The occurrence of SP was significantly associated with the season. Indeed, the highest number of pneumothorax was noted during the summer with 61 cases (32.4%) compared to 50 cases in spring (26.6%), 47 cases in autumn, and only 30 cases (16%) in winter (p = 0.015) (Figure 1).

Comparing the onset of pneumothorax during summer to the other seasons, a higher risk was noted with an OR = 1.44 (95% CI [1.04-1.99]) and p = 0.0027. This association was greater while comparing warm seasons (spring-summer-autumn) and cold season (winter), with a risk of occurrence of SP during the relatively warm seasons 1.81 times greater than in winter (p = 0.004, OR = 1.81 (95% CI [1.2-2.72])).

### 3.4. Meteorological Parameters Effects on the Onset of Spontaneous Pneumothorax

#### 3.4.1. Mean Temperature

The comparison of mean temperatures between days with and without pneumothorax showed significantly higher temperatures during the days of occurrence of this disease with p = 0.021. The same observation was noted by comparing the mean temperatures one day and two days before the onset of pneumothorax with p = 0.006 and p = 0.011, respectively.

**Figure 2:**
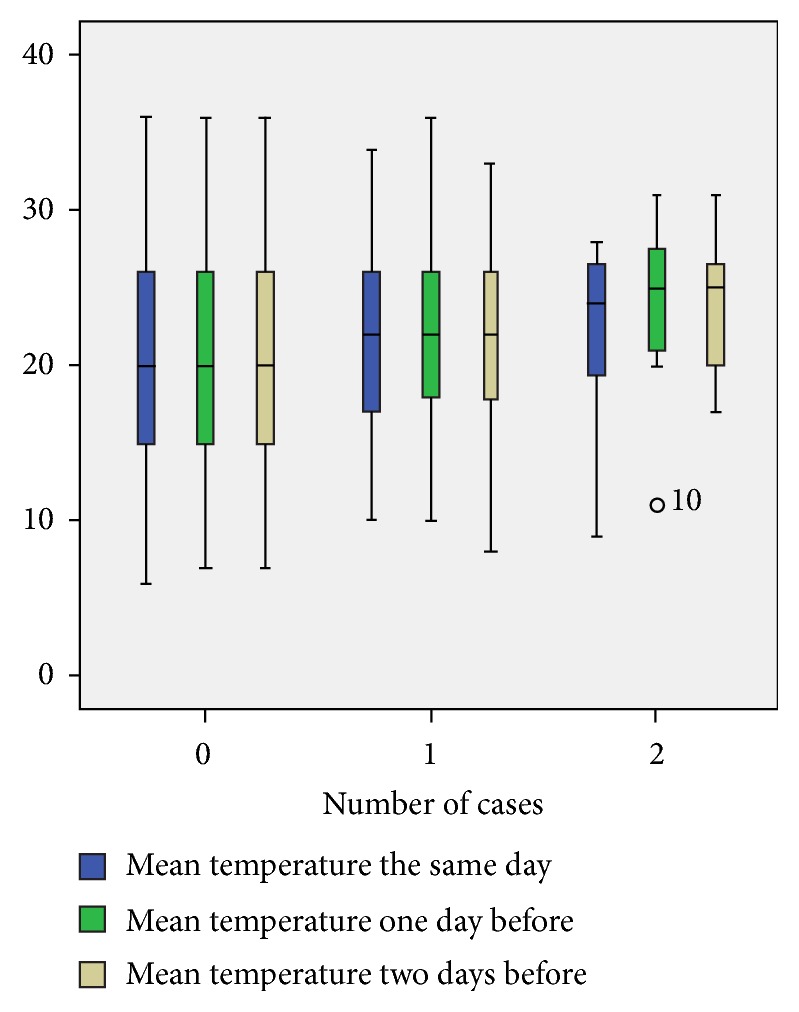
Association between the number of cases of pneumothorax and the mean temperature.

Figure 2 shows the distribution of the different average temperatures according to the number of cases observed.

Poisson GLM regression showed a significant increased risk of occurrence of a pneumothorax of 3.4% for each temperature rise of 1°C. This association was observed for temperatures one day and two days before the occurrence of pneumothorax with increased risks of 3.8% and 3.6%, respectively, for each 1°C.

Considering the collinearity of these three variables (average temperature on the day of occurrence of pneumothorax, a day before and two days before), mean temperatures of all three days were calculated. Thus, an increase of mean temperature of the three days by 1°C augmented the risk of the onset of pneumothorax by 3.7% (p = 0.003; exp⁡(B) = 1.037, 95% CI [1.013-1.062]).

#### 3.4.2. Mean Relative Humidity

**Figure 3:**
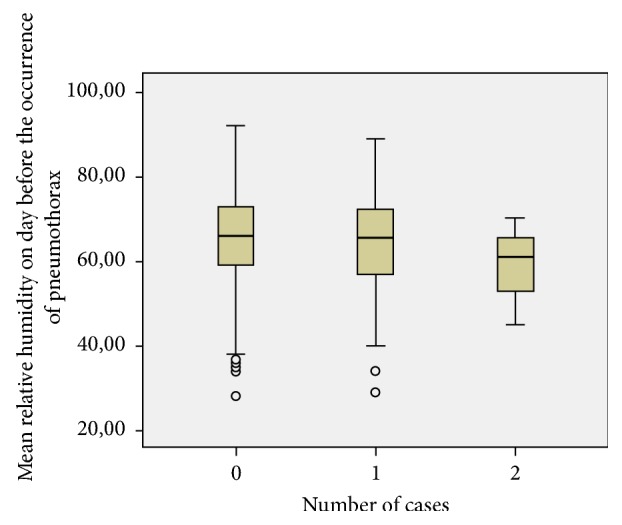
Association between mean humidity and the number of cases of pneumothorax.

Although mean relative humidity between days with and without pneumothorax did not differ significantly (p = 0.33), a lower relative humidity one day before the occurrence of pneumothorax was related to the risk of its onset. The comparison of mean relative humidity averages one day before pneumothorax showed that this fall was similarly associated with the number of pneumothoraxes the next day with p = 0.036 as shown in Figure 3.

The mean relative humidity two days before the occurrence of pneumothorax did not appear to be associated with the risk of occurrence of pneumothorax (p = 0.26).

Poisson regression showed that the decrease in mean relative humidity one day before of 1% was associated with an increased risk of developing a pneumothorax the next day by 1.6% (p = 0.02, exp⁡(B) = 0.984, 95% CI [0.97-0.99]).

#### 3.4.3. Mean Atmospheric Pressure

Mean atmospheric pressure the same day, one day before, and two days before the occurrence of pneumothorax did not appear to be associated with an increased risk of pneumothorax.

#### 3.4.4. Mean Duration of Sunshine

**Figure 4:**
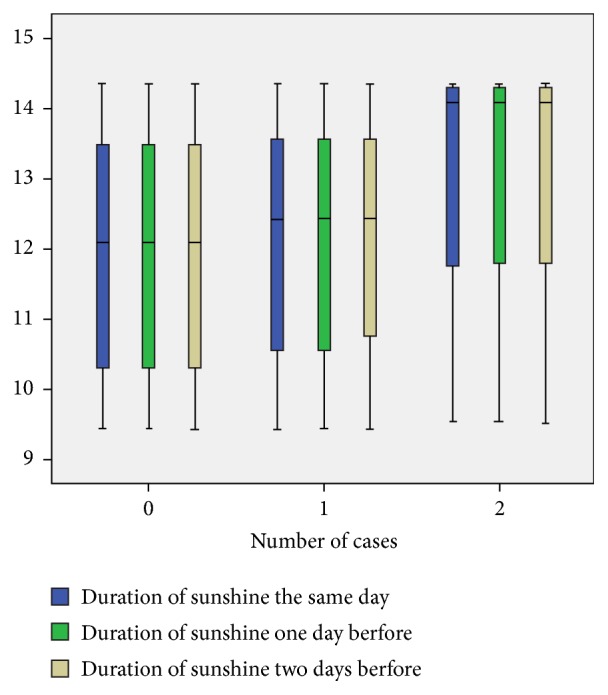
Distribution of mean duration of sunshine in the same day, one day before, and two days before the onset on pneumothorax according the number of cases of pneumothorax.

The mean of duration of sunshine the same day, one day before, and two days before the occurrence of pneumothorax was statistically associated with the onset of this pathology with p = 0.008 at the same day, p = 0.006 one day before, and p = 0.005 two days before. Indeed, this mean duration of sunshine was higher on the same day of pneumothorax, as well as one day, and two days before, compared to days without pneumothorax (Figure 4).

Moreover, the longer this duration was, the higher the number of cases was. Taking into account the collinearity of the mean duration of sunshine the same day, one day before, and two days before, Poisson regression analysis was performed considering the average of all three days. Thus, an increase of one hour in the average duration of sunshine during these 3 days was associated with an increased risk of pneumothorax of 14% (P = 0.002, exp⁡(B) = 1.14 95% CI [1.04 to 1.24]).

#### 3.4.5. Rainfall and Thunderstorms

In our study, 17.6% of days of pneumothorax were rainy (n = 321 days). Rainfall occurring the same day, one day before, and two days before did not seem to be associated with a higher risk of pneumothorax (p = 0.98, 0.83, and 0.7 respectively).

During the study period, 142 days included the occurrence of thunderstorms which were not statistically associated with the occurrence of pneumothorax.

### 3.5. Main Results

A higher risk of occurrence of pneumothorax during summer was noted with an OR = 1.44 (95% CI [1.04-1.99]) and p = 0.0027. This association was greater while comparing warm seasons (spring-summer-autumn) and cold season (winter), with a risk of occurrence of SP during the relatively warm seasons 1.81 times greater than in winter (p = 0.004, OR = 1.81 (95% CI [1.2-2.72])).

We showed a significant increased risk of occurrence of a pneumothorax of 3.4% for each temperature rise of 1°C. This association was observed for temperatures one day and two days before the occurrence of pneumothorax with increased risks of 3.8% and 3.6%, respectively, for each 1°C.

We noted that the decrease in mean relative humidity one day before of 1% was associated with an increased risk of developing a pneumothorax the next day by 1.6% (p = 0.02, exp⁡(B) = 0.984, 95% CI [0.97-0.99]). An increase of one hour in the average duration of sunshine during these 3 days was associated with an increased risk of pneumothorax of 14% (P = 0.002, exp⁡(B) = 1.14 95% CI [1.04 to 1.24]).

### 3.6. Other Analyses

#### 3.6.1. *«* Clusters *»* and Meteorological Parameters

In our study, the occurrence of clusters was associated with higher mean temperature in the same day, one day before and two days before with p = 0.038, p. = 0.008, and p = 0.006, respectively.

This same observation was made concerning the mean duration of sunshine two days before the occurrence of the cluster which was higher with p = 0.05.

In addition, the occurrence of thunderstorms two days before the occurrence of clusters was significantly associated with a risk multiplied by 1.96 (p = 0.035, OR = 1.96, 95% CI [1.038-3.69]).

#### 3.6.2. Multivariate Analysis

*Onset of SP and Variables of Interest. *A multiple binary logistic regression was performed to determine the independent variables associated with the occurrence of a pneumothorax by transforming meteorological data into binary qualitative variables based on their quartiles. A selection of the most significant quartiles for a univariate analysis was previously performed. This preliminary analysis indicated that the occurrence of pneumothorax was significantly associated with an average temperature greater than or equal to 16°C (1st Quartile) with p = 0.0022 and OR = 1.58 (95% CI [1.06 -2.33]). This association was also noted for temperatures greater than or equal to 20°C with p = 0.003 and OR = 1.6 (95% CI [1.17-2.2]). Thus, the increase in temperature from Q1 to Q2 (the median temperature) was associated with an increased risk of pneumothorax of 2%.

After multiple binary logistic regression, only a mean temperature above the median of 20°C was associated with higher risk of pneumothorax occurrence by 60%.

*Onset of « Clusters » and Variables of Interest. *Multiple binary logistic regression showed that the occurrence of “clusters” was significantly associated with the occurrence of thunderstorms two days before the occurrence of pneumothorax and the summer season. Thus, clusters would be 1.74 times more frequent in summer and their occurrence would be increased by 94% in case of occurrence of thunderstorms two days before their incidence.

## 4. Discussion

During the 5th century BC, Hippocrates thought that climate could play a role in worsening the health of people [13]. Many studies have suggested the possible intervention of weather conditions such as temperature, humidity, atmospheric pressure, or precipitations on the occurrence of several diseases. Among these associations, mention was made of gout, asthma, Behçet's disease, or the rupture of the abdominal aortic aneurysm [14, 15].

The effect of a sudden change of pressure on the occurrence of pneumothorax is still discussed. Several studies have been conducted to find a relationship.

Cran IR. and Rumbal CA. noted that the incidence of pneumothorax was higher among Royal Air Force personnel, which was an argument in favor of this theory [16]. In addition, Ohata M. and Suzuki H. showed in their studies that the atmospheric pressure had an effect on the air leakage within the pleural cavity through the emphysema bubble [17].

If the onset of SP was influenced by weather changes, it should be common in various locations throughout the world, with various climates. There have been some reports referring to correlations between the risk of pneumothorax and weather conditions but these were mostly from Europe and the United States [9, 11, 18–22].

### 4.1. Keys Results

Tunisia's climate has some specificities and to our knowledge, there are no Tunisian studies published on this subject. This study confirmed the well-established finding that men are much more susceptible to spontaneous pneumothorax than women probably due to higher smoking rates, greater height, and relatively smaller airways among men [1, 23–31].

In our study, young people without significant lung pathology were the most affected. The mean age was 28 ± 17.11 years and the most represented age group was between 21 and 40 years old (58%). Similar results have been found in the literature [20, 21, 25–27, 32].

### 4.2. Strengths and Limitations

This study has several strengths. First, it is the first study examining the effects of seasonal variations and changes in meteorological parameters on the occurrence of pneumothorax in a Mediterranean climate as in Tunisia. Sousse is the region that occupies the eastern center of Tunisia. It covers an area of 2 669 km^2^ (1.6% of the country's area). It is located on the Tunisian coastline. The region of Sousse has a Mediterranean coastal climate characterized by hot, dry summers, and mild, wet winters [31] .

Second, we carried out a study of a time series, including the collection of meteorological parameters over a period of 5 years with analysis of their possible relationship with the onset of SP, or the successive occurrence of several cases of pneumothorax in a short period. This method would be the method of choice to study the short-term relationship between meteorological parameters and acute health events, compared to other methods such as cross-case studies where we compare two periods of time and cases that will be their own control [33].

There are, however, some limitations to this study. In fact, during the study period, 200 cases of SP were collected after verification of the inclusion and exclusion criteria. The cases have been identified in the university hospitals where the management of SP is carried out in the region of Sousse. However, this number could be unrepresentative given the possibility of the management of this pathology in private clinics making the collection of cases not exhaustive.

### 4.3. Interpretation and Generalisability

Neither particular year nor seasons were found to be significantly associated with a higher risk of onset of SP. Celik B. et al. in Turkey have found that more cases occurred mainly in autumn with no statistical difference with other seasons. [25] Bulajich B. et al. in Belgrade showed a maximum number of cases of pneumothorax during winter, also with no significant difference with the other seasons [22].

For Suarez-varela M., the percentage of cases during the spring was the largest (33.87%) but without being significant [21]. Contrary to these findings, several studies have found no association between SP cases and seasons [11, 20, 34].

Ozenne G. et al. have showed two frequency peaks: in summer and in winter with a significant association with humidity [35]. These authors explained that bronchoconstriction is induced by the humidity of the air in the airways and that this could play a role in the occurrence of pneumothorax [9].

The temperature of the air entering the human body is balanced with body temperature by crossing the mouth or nose and the airways to the alveolar space. The presence of an association between rising temperatures and the occurrence of pneumothorax is cited in several studies and cannot be ignored.

According to Celik B., Heyndrickx M., Smit HJ., Schieman C., and Haga T, there was no significant difference, comparing meteorological data between days without and with pneumothorax [20, 25, 26, 29, 36].

However, other studies showed that an increase in mean temperature increased the risk of pneumothorax and supported our findings [9, 20, 28, 37].

Our results are comparable to those of a recent study conducted by Motono N. et al. in 2016, which noted an influence of mean temperature on the occurrence of spontaneous pneumothorax. Blebs and bubbles with a nonreturn valve would trap air. This air behaved according to the law of Boyle-Marriot (Pressure *∗* Volume = Constant). Increased temperatures would participate in the expansion of the gases and therefore a risk of breaking the blebs. Although the air contained in these blebs is not influenced by the external temperature, the latter influences the atmospheric pressure which would lead to the same result [38].

Among the mechanisms responsible for the development of SP, we thought that, when the air inside the bleb bubble was trapped because of bronchospasm, this would cause an imbalance between the pressure gradient with the environment [20, 34]. For others, the transpulmonary pressure gradient may be sufficient to cause pneumothorax following a fall in atmospheric pressure [9]. According to the literature, it has been argued that the change in atmospheric pressure could be the cause of a pneumothorax since this has been especially noticed by airline personnel [16].

Some authors believe that a sudden change in atmospheric pressure may lead to a SP. According to Bertolaccini L., there is no relationship between the occurrence of pneumothorax and atmospheric pressure changes. One might think that a longer exposure to these variations may be necessary to provoke the event. However, Scott GC. et al. only described the hospitalization of only one among four patients after a 4-day exposure to changes in atmospheric pressure [19, 39].

In our study, mean atmospheric pressure levels the same day, one day before, and two days before did not seem to be associated with a higher risk of pneumothorax.

In regard to the relative humidity, studies often suggest the lack of a relationship between relative humidity and the occurrence of SP. [25, 26, 28, 29, 40]

However, Ozenne G. et al. reported lower relative humidity during the days of pneumothorax [35]. These results were similar to those found in our study where the lower relative humidity, one day before the occurrence of SP, the greater the number of pneumothorax (p = 0.036).

The explanation given by Ozenne G. et al. is that the decrease in relative humidity is accompanied by humidification of the airways with secondary bronchoconstriction, precipitating the onset of SP [35].

Regarding other meteorological parameters, Alifano et al. have noted the onset of clusters during or immediately following a fall in atmospheric pressure [11].

There is no consensus regarding the reasons for the apparent increase in the rate of SP or the factors precipitating its development. However, physicians have observed that patients with SP are admitted in clusters and changes in climate or related weather conditions are therefore suspected to be involved in the occurrence of SP.

Smit HJ. was the first to note that 73% of primitive pneumothoraxes were admitted as clusters. Based on his clinical findings, he defined the cluster as the hospitalization of two or more patients for SP with less than 3 days without episodes between the pneumothorax events (95%, p <0.001) [20].

This was also the conclusion of Boulay F. et al. who noted the occurrence of 60% of SP cases as clusters, with an average of 2.7 patients in each cluster [20, 36, 41, 42].

In a study carried out in Turkey between 1996 and 2006, 669 episodes of SP were noticed and 472 appeared in 188 “clusters”. The average clustered case was 2.5 ± 0.8 with a minimum of 2 episodes and a maximum of 7 [9].

For Vodicka J., 23.3% of the cases were observed in 54 “clusters”. This low rate was explained by the low number of cases recorded over a long period of time (22 years) [37].

Celik B. et al. found a relationship between these clusters and the increase in mean temperature (p = 0.038), and this result was similar to ours [25]. However, we noticed the association between the occurrence of storms two days before the onset of SP and these clusters, which was not found by other authors.

## 5. Conclusion

In summary, we conclude that there is an association between the occurrence of SP and some metrological parameters.

Other studies have shown different results from ours and this could be explained by the differences that exist between regions in terms of climatic specificities and meteorological conditions.

Our study draws attention to this relationship that exists between seasonality and the onset of SP in the region of Sousse.

To our knowledge, it is the first study that was conducted in the southern side of the Mediterranean region to prove this correlation.

Our main result is the association between warm season, high mean temperature, and SP. This observation would explain the onset of clusters and allows a scientific approach to the “law of series” that clinicians note.

Expecting a higher number of cases during hot seasons, physicians can only be better prepared to manage them.

## Figures and Tables

**Table 1:** Demographic and clinical characteristics of patients with spontaneous pneumothorax in Sousse, Tunisia, 2010-2014 (n=200).

Variable		n(%)
Gender	(i) Male	191(95.5)
	(ii) Female	9(4.5)

Respiratory medical history	Asthma	2(1)
	COPD	12(6)
	Interstitial lung disease	1(0,5)

Non respiratory medical history		50(25)

No medical history		137(67.5)

Smoking		162 (81)

Localization of the pneumothorax	Right	100 (50
	Left	99(49.5)
	Bilateral	1(0.5)

Nature of pneumothorax	Spontaneous idiopathic pneumothorax	151(75.5)
	Spontaneous secondary pneumothorax	49(24.5)

Abundance of pneumothorax	Partial pneumothorax	53(26.5)
	Total pneumothorax	147(73.5)

**Table 2:** Mean values for meteorological variables in Sousse, Tunisia, 2010-2014.

Variable	Mean	SD	Minimum	Maximum
Ambient temperature (°C)	20.6°C	6.01°C	6	36

Relative humidity (%)	65.55	9.9	28	92

Atmospheric pressure (hPa)	1015.43	5.31	996.98	1032.22

Rainfall (mm)	321			

Sunshine (h)	11.97	1.69	9.44	14.35

## Data Availability

The meteorological data used to support the findings of this study are available from the corresponding author upon request.

## Supplementary Materials

Supplementary Materials The meteorological data and patients data are saved in an SPSS document (attached), the document for data collection in word version.
