# Supplementary material for: Influence of Weather Conditions on the Onset of Spontaneous Pneumothorax in the Region of Sousse (Tunisia): Analysis of Time Series
Source: Biomed Res Int. 2019 May 7;2019:1793973. doi: 10.1155/2019/1793973 (PMC6530201; doi:10.1155/2019/1793973)

**Meteorological data**

Date :…………………………………………

- Mean temperature : …………………….. °C
- Mean temperature on day lag :…………….. °C
- Mean temperature 2 days lag :…………….. °C
- Mean relative humidity :………………..%
- Mean relative humidity one day lag:………..%
- Mean relative humidity 2 days lag :……..%
- Mean atmospheric pressure :………………hPa

• Mean atmospheric pressure one day lag :………………hPa

• Mean atmospheric pressure 2 days lag :………………hPa

- Duration of sunshine  :…………. Hours
- Duration of sunshine   one day lag :…………….hours
- Duration of sunshine  2 days lag :…………….hours
- Occurrence of thunderstorms : yes
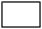
 No
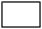

- Occurrence of precipitations : yes
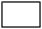
 No
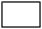


- Number of cases of pneumothorax :…………………..

Record card of Patient’s data

Number of file :

| Patient  Name :  Fist name : |
| --- |

Department :

Age :

Date of birth :

Sex : F
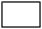
 M
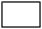


Smoking habits :

Cigarettes
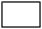


Shisha
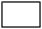


Localization : right
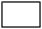


left
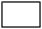


Bilateral
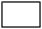


Type of pneumothorax : Idiopathic
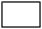


Secondary
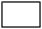


Partial
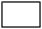


Total
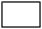


Respiratory medical history :

Antécédents respiratoires : COPD
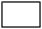
 Interstitial desease
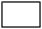


Asthma
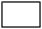
 others
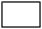


Pleural talcage : yes
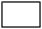
 No
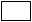


Treatment: Rest
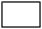
 Oxygen
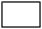


Exsufflation
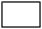
 chest drain
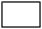


Evolution : Favorable
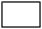
 worsening
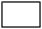


Date of occurrence of pneumothorax :

Recidivism : yes
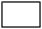
 No
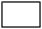

Supplement: Supplementary Materials — The meteorological data and patients data are saved in an SPSS document (attached), the document for data collection in word version. [file 1793973.f1.doc]
